# Factors associated with multiple‐death suicides among rural US decedents

**DOI:** 10.1111/jrh.70003

**Published:** 2025-02-17

**Authors:** Victor A. Soupene, Timothy Hays, Jonathan Davis, J. Priyanka Vakkalanka

**Affiliations:** ^1^ Department of Emergency Medicine University of Iowa Health Care Iowa City Iowa USA; ^2^ Department of Epidemiology University of Iowa College of Public Health Iowa City Iowa USA; ^3^ Department of Occupational and Environmental Health University of Iowa College of Public Health Iowa City Iowa USA

**Keywords:** public health surveillance, qualitative research, rural health, social determinants of health, suicide

## Abstract

**Purpose:**

To identify contributing circumstances to multiple‐death suicides in rural US counties from law enforcement and coroner/medical examiner narratives.

**Methods:**

We identified multiple‐death suicides as multiple suicides (i.e., two or more decedents) from the National Violent Death Reporting System (NVDRS) between 2013 and 2021. We identified rural decedents from their residence in NVDRS using federal information processing codes linked to Rural–Urban Continuum Codes. Quantitatively, we described demographic characteristics and circumstances leading to the suicide. From narrative law enforcement and coroner/medical examiner reports, we generated codes describing demographic and circumstance information using Dedoose software. We then used inductive content analysis to identify themes related to multiple‐death suicides.

**Findings:**

Among all multiple‐death suicides (*n* = 50 multiple‐death suicides, 99 suicide decedents), decedents were mostly non‐Hispanic White (*n* = 90; 91%), were male (*n* = 54; 54%), and had a high school diploma or less (*n* = 51; 56%). We identified four themes: meticulous and considerate planning (e.g., with notes, reasons, and next steps), mutual dependency (e.g., decedents were in separable), unbearable health conditions (e.g., pain, poor quality of life from chronic illnesses), and social factors of despair (e.g., financial strain, legal problems, and interpersonal violence).

**Conclusion:**

Developing strategies for discussing suicidal ideation and improving access to financial resources and health care may reduce multiple‐death suicides in the rural United States, particularly among older adults with chronic health problems. Improving other public health initiatives such as interpersonal violence and chronic disease prevention and management may further prevent multiple‐death suicides.

## INTRODUCTION

Understanding the etiology of suicide is critical as it is a significant public health concern; suicide is the 11th leading cause of death overall in the United States^1^ but is one of the top five causes of death for those 10–44 years of age.^2^ Suicide rates vary by race, ethnicity, and gender, but more recently, the Centers for Disease Control and Prevention (CDC) has reported that age‐adjusted rates were disproportionately greater in rural (16.3 per 100,000) compared to urban (11.9 per 100,000) US areas during 2001–2022.^3^ While suicide rates increased by 27% in urban US areas, rural US areas have experienced an increase of 46% between 2000 and 2020.^4^, ^5^ Rural US areas have also been inequitably afflicted by risk factors for suicide, including substance use,^6^, ^7^, ^8^ economic distress,^6^, ^8^ and significant barriers to accessing health care,^6^, ^9^, ^10^, ^11^, ^12^, ^13^ which have been expected to further exacerbate suicide rates in the rural United States.

Most studies examining suicide and risk factors have focused on single suicides.^6^ Less commonly occurring, though significant in impact, is the occurrence of multiple‐death suicides that are suicides involving two or more individuals who are linked to each other.^14^, ^15^, ^16^, ^17^ Examples of multiple‐death suicides are “suicide pacts,” defined as two or more individuals agreeing to die together,^15^, ^17^ and “suicide contagions,” defined as one suicide that directly or indirectly contributes to another suicide.^18^ Multiple‐death suicides are arguably more devastating to friends, family, and communities of the decedents given that multiple deaths are involved.^15^ However, few studies have examined how multiple‐death suicides differ from single suicides.^15^, ^16^ Previous studies have observed that multiple‐death suicides compared to single suicides were more likely to involve older adults (55 years and older) who were non‐Hispanic White and married.^15^, ^16^ Risk factors that were more likely to contribute to multiple‐death suicides compared to single suicides included having physical health problems, having financial problems, and opioid use.^15^, ^16^ While these studies identify certain demographic characteristics and risk factors that increase the risk of multiple‐death suicides compared to single suicides,^15^, ^16^ both studies lack detailed descriptions of how these risk factors contribute to multiple‐death suicides and whether factors related to one's residence (i.e., living in a rural area) may also affect multiple‐death suicides. It is unclear whether current prevention strategies for addressing suicide in rural communities are also applicable to multiple‐death suicides.

Given the increase in suicides in the rural United States and the need to understand what factors, in detail, contribute to multiple‐death suicides, our goal was to explore the unique characteristics of multiple‐death suicides in the rural United States. This study aims to provide both quantitative and qualitative insights into multiple‐death suicides, offering a comprehensive assessment of this phenomenon in rural contexts. We hypothesized that rural‐related factors including limited access to health care increased the odds of multiple‐death suicides compared to single suicides in the rural United States.

## METHODS

### Study design, setting, and sample

We conducted a mixed‐methods study using circumstance and narrative data from the National Violent Death Reporting System (NVDRS) during 2013–2021. NVDRS is a case‐only, national dataset that contains information on violent deaths (e.g., suicide) from coroner or medical examiner (CME) reports, law enforcement (LE) reports, death certificates, and toxicology reports.^14^ We used data for deaths categorized as “multiple suicides,” defined as two or more suicide deaths that are linked into a single incident.^14^ Per NVDRS guidance,^14^, ^16^ we also included deaths categorized as “multiple deaths—other” and individually examined each to determine if they could be included. However, we determined that none of the cases that met our inclusion criteria should be included, given the uncertainty in whether they were all suicidal deaths. Decedents with no known circumstances were not included in the analysis, as suggested by NVDRS guidelines.^14^

We linked Rural–Urban Continuum Codes (RUCCs) to NVDRS incidents using federal information processing system codes and only included those that are considered nonmetropolitan (RUCC = 4–9).^19^ This study was reviewed by our local Institutional Review Board, which determined this was not human subjects’ research.

### Quantitative and qualitative data elements

We identified demographic characteristics of all suicide decedents resulting from multiple‐death suicides, including sex, age group, race/ethnicity, education level, marital status, region of the United States where the decedent lived, and lethal mean used. We further assessed situational circumstances including having financial problems, having criminal legal problems, having family relationship problems, having current or former intimate partner problems, having a suicide note present, disclosure of suicide intent, a history of attempting suicide, a history of mental health/substance use treatment, having a current physical health problem, having a current mental health problem, having a current alcohol use problem, and having a current non‐alcohol‐related substance use problem. While the NVDRS data have extensive information about circumstances that could contribute to suicide, it is not exhaustive of every factor that can contribute to suicide risk. To identify additional factors specific to multiple‐death suicides, we used the abstractor narrative summary of the medical examiner and/or law enforcement report to identify additional factors that contributed to multiple‐death suicides among rural US residents.

### Analysis

We conducted descriptive analyses of demographic characteristics including the age group at the time of their death (18–24, 25–44, 45–64, and >65 years), biological sex (male, female), race/ethnicity (White, non‐Hispanic; not White, non‐Hispanic), education level (high school graduate or less, some college or associate's degree, college degree or more), marital status at the time of their death (married/civil union/domestic partnership, separated/widowed, single), lethal mean used (firearm, poisoning, suffocation, or other [e.g., motor vehicles, fires/burns, and sharp instruments]), and the region of the United States the decedent lived in at the time of their death. US regions included the Northeast (Connecticut, Maine, Massachusetts, New Hampshire, New Jersey, New York, Pennsylvania, Rhode Island, Vermont), Southeast (Alabama, Arkansas, Delaware, Florida, Georgia, Kentucky, Louisiana, Maryland, Mississippi, Missouri, North Carolina, Oklahoma, South Carolina, Tennessee, Texas, Virginia, West Virginia), West (Alaska, Arizona, California, Colorado, Hawaii, Idaho, Montana, Nevada, New Mexico, Oregon, Utah, Washington, Wyoming), and Midwest (Illinois, Indiana, Iowa, Kansas, Michigan, Minnesota, Missouri, Nebraska, North Dakota, Ohio, South Dakota, Wisconsin). Due to data suppression rules (i.e., cannot report for variables with less than <10 decedents), race/ethnicity could not be reported in the table (around 90% multiple‐death suicide decedents were White, non‐Hispanic).^20^

We conducted qualitative analyses using abstractor summaries of CME and LE narratives, using Dedoose Version 9.2.12.^21^ We followed an inductive thematic analysis procedure, meaning we read each narrative in detail and assigned codes as we read the narratives. After the initial coding step, we developed a codebook to reduce the number of original codes into larger groupings. The codebook includes the codes along with their respective definitions and boundaries (Appendix A). We then combined codes to create themes. The themes were finalized after discussion among the research team. The first and second authors undertook the coding process, and the first author generated the themes. All qualitative analyses were conducted using Dedoose Version 9.2.12.^21^ All quotes used were composites of the original narrative text combined together, and pronouns were changed to gender‐neutral terms (they/them/theirs) to promote anonymity, as requested by the NVDRS guidelines.^20^

We conducted quantitative analyses using circumstances in NVDRS related to identified themes and factors previously shown to be associated with suicides in the rural United States.^6^, ^22^ To examine the association between demographic characteristics and multiple‐death suicides compared to single suicides in the rural United States, we developed univariable logistic regression models. We then developed multivariable logistic regression models to examine the association between each circumstance and multiple‐death suicides compared to single suicides in the rural United States. We determined a priori that we would include age, race/ethnicity, biological sex, educational level, and marital status in each multivariable model. We reported odds ratios (ORs) with 95% confidence intervals (CIs) for each variable and conducted all quantitative analyses using SAS (SAS Institute Inc).

### Reflexivity

Because of our positionality as researchers,^23^ we find it important to provide details about our study team and our roles in the process of conducting qualitative research. The first author is a male, PhD candidate in a department of epidemiology who studies rural health outcomes and is from a rural US area. The first author was supported by the second and third authors in conducting the qualitative and quantitative research analyses. The second author is a medical student who was engaged in coding the narratives and composing the themes. The third author is a research faculty member in a department of occupational and environmental health who assisted with the quantitative analysis. The senior author is a female, research faculty member in a department of emergency medicine who studies rural health factors related to suicide and drug overdoses. The senior author directed the study and assisted in clarifying the thoughts around multiple‐death suicides among rural adults.

## RESULTS

### Descriptive statistics

Between 2013 and 2021, there were 54 multiple‐death suicide incidents that occurred in the rural United States with a total of 108 suicide decedents (Table 1). Of these decedents, at least one decedent in four of the multiple‐death suicide incidents did not have descriptive statistics reported and were excluded, resulting in 50 multiple‐death suicide incidents and 99 suicide decedents. Multiple‐death suicide decedents had higher odds of being older than 65 years compared to <45 years (unadjusted OR = 3.09, 95% CI: 1.93, 4.94), being female compared to male (unadjusted OR = 3.44, 95% CI: 2.32, 5.12), and being married, in a civil union, or in a domestic partnership compared to being single (unadjusted OR = 3.18, 95% CI: 1.92, 5.27) (Table 1). They had lower odds of being a high school graduate or less (unadjusted OR = 0.39, 95% CI: 0.24, 0.64) or completing some college or an associate's degree (unadjusted OR = 0.42, 95% CI: 0.23, 0.79) compared to having a college degree or more. Decedents in multiple‐death suicides were at higher odds of dying from a poisoning‐related suicide (unadjusted OR = 5.06, 95% CI: 3.21, 7.96) or other suicide (unadjusted OR = 3.92, 95% CI: 2.05, 7.50) compared to firearm‐related suicides (Table 1).

**TABLE 1:** Descriptive characteristics of suicide decedents identified in the rural United States, NVDRS 2013–2021.

	Total (*n* = 46,566)	Multiple‐death suicides (*n* = 99)^a^	Single suicides (*n* = 45,467)	Univariable model
Characteristic	*N* (%)	*n* (%)	*n* (%)	Unadjusted odds ratio (95% CI)
Sex				
Female	8237 (20)	45 (46)	8192 (20)	3.44 (2.32, 5.12)
Male	33,883 (80)	54 (54)	33,829 (80)	Ref
Age				
45–64 years	14,164 (34)	27 (27)	14,137 (34)	1.22 (0.73, 2.06)
>65 years	10,207 (24)	42 (42)	10,165 (24)	**3.09 (1.93, 4.94)**
<45 years	21,501 (46)	30 (30)	19,193 (46)	Ref
Education level				
High school graduate or less	26,546 (67)	51 (56)	26,495 (67)	**0.39 (0.24, 0.64)**
Some college or associate's degree	8668 (22)	18 (20)	8650 (22)	**0.42 (0.23, 0.79)**
College degree or more	4482 (11)	22 (24)	4460 (11)	Ref
Marital status				
Married/civil union/domestic partnership	13,841 (33)	62 (63)	13,779 (33)	**3.18 (1.92, 5.27)**
Separated/widowed	13,759 (33)	17 (17)	13,742 (33)	0.88 (0.46, 1.67)
Single	14,167 (34)	20 (20)	14,147 (34)	Ref
Region				
Northeast	3648 (9)	12 (12)	3636 (9)	**2.03 (1.00, 4.10)**
Southeast	15,796 (38)	39 (39)	15,757 (38)	1.52 (0.90, 2.57)
West	9046 (22)	26 (26)	9020 (22)	**1.77 (1.00, 3.13)**
Midwest	13,536 (32)	22 (22)	13,514 (32)	Ref
Lethal mean used				
Poisoning	4616 (11)	36 (36)	4580 (11)	**5.06 (3.21, 7.96)**
Suffocation	10,395 (25)	12 (12)	10,383 (25)	0.74 (0.39, 1.42)
Other	1982 (5)	12 (12)	1970 (5)	**3.92 (2.05, 7.50)**
Firearm	25,127 (60)	39 (39)	25,088 (60)	Ref

*Note*: Bolded *p*‐values <0.05.

Abbreviation: NC, not calculated due to data suppression (*n* < 10).

^a^ Missing descriptive statistics for at least one suicide decedent in the multiple‐death suicides for nine of the observations.

**FIGURE 1:**
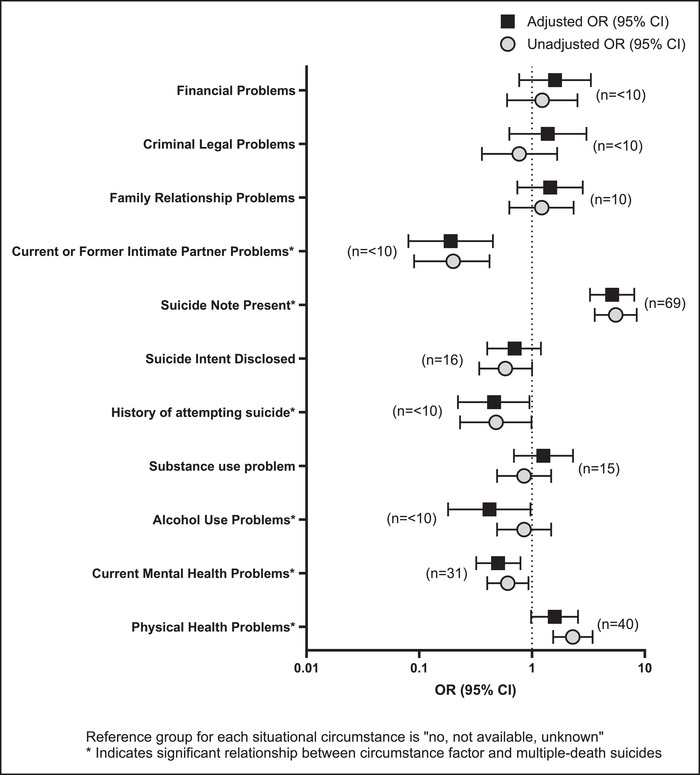
Relationship between situational circumstances and multiple‐death suicides (*n* = 99) compared to single suicides (*n* = 45,467) among rural US decedents (*n* = 46,566); National Violent Death Reporting System 2013–2021.

In the adjusted models, decedents who wrote suicide notes had higher odds of dying from multiple‐death suicides compared to single suicides in the rural United States (adjusted OR = 5.14, 95% CI: 3.37, 8.08) (Figure 1). Multiple‐death suicide decedents also had lower odds of having alcohol use problems (adjusted OR = 0.42, 95% CI: 0.18, 0.97), mental health problems (adjusted OR = 0.50, 95% CI: 0.32, 0.79), current or former intimate partner problems (adjusted OR = 0.19, 95% CI: 0.08, 0.45), and a history of attempting suicide (adjusted OR = 0.46, 95% CI: 0.22, 0.95) at the time of their death compared to single suicides in the rural United States. Having physical health problems was not significantly different between multiple‐death suicides and single suicides in the rural United States in the adjusted model.

### Qualitative analyses

We identified four themes pertaining to factors contributing to multiple‐death suicides in the rural United States: (1) meticulous and considerate planning; (2) mutual dependency; (3) unbearable health conditions; and (4) social factors of despair (Table 2).

**TABLE 2:** Summary of themes from qualitative analysis with definitions.

Theme	Definition
Meticulous and considerate planning	Multiple‐death suicides often were planned ahead of time by both victims to ensure the suicide was completed. Planning also entailed planning out their wills and providing their children or family members with their financial information, plans for their deceased bodies, and notes for why they were dying by suicide.
Mutual dependency	Multiple‐death suicides often involved intimate partners or close family members and friends. Many of these suicide decedents had disclosed or described in suicide notes their dependency on the other victim or their inability to live without this other person.
Unbearable health conditions	Physical and mental health conditions and substance use contributed to multiple‐death suicide decedents, with most afflicting older adults. These health conditions made it difficult for many suicide decedents to live happy and healthy lifestyles. Many suicide decedents disclosed to family and friends or through their suicide notes that they wanted to die “on their own terms” or not burden their family and friends with their health conditions.
Social factors of despair	Social factors such as having financial problems, legal problems, and being victims of violence were common among multiple‐death suicides. Decedents may have struggled with payments for housing, may have had criminal allegations of abuse toward them or were otherwise facing jail time, or may have been bullied by their peers. These factors were implied to have contributed to their decision to attempt and complete suicide.

### Meticulous and considerate planning

We defined this theme by the level of planning by both decedents, which often consisted of will planning, plans for outstanding logistical tasks, and notes identifying reasons for the planned suicide. For example, law enforcement officers “located a file including a check for their roommate for overpaying rent, a suicide note instructing their next of kin how to take care of their estate, and their will.” Planning also included funeral arrangements and next steps for loved ones, “both victims left suicide notes detailing locations of money and vehicle titles… one victim had made arrangements for both funerals and paid for both prior to their deaths.” The notes often indicated the decedents did not want to become a burden to loved ones and included apologies to the recipient. In one instance, one family member of the decedents who received a suicide note reported “both victims told them they were going to die by suicide once they felt like they were burdening them. The family member said they were aware that they had purchased some pills for their suicide. The suicide notes were found at the scene showing that the suicides were planned together.”

### Mutual dependency

We found that a common aspect included mutual dependency, defined from notes as the dependency on the other victim or their inability to live without this other person. While this could have included close family members or friends, we found that most often it involved decedents who were spouses or intimate partners. Some notes included that the decedents “were inseparable and didn't want to live without each other…both decedents agreed to take their lives together,” and “Victim 2 found Victim 1 and decided they couldn't live without Victim 1 and then took her own life.” Children of decedents echoed these sentiments saying, as one family member indicated “[their parents] were married for over 30 years and ‘lived for each other.’”

### Unbearable health conditions

One commonly observed theme among rural multiple‐death suicides in this study included the presence of physical and mental health conditions that made it difficult for suicide decedents to live happy and healthy lifestyles. These often presented as disclosures to family and friends of wanting to die on their own terms. Physical conditions included a wide range of health concerns, including, but not limited to, terminal cancer, multiple sclerosis, pain, and dementia. For example, one suicide note explained “victim 1 was struggling with dementia, victim 2 had other health issues and could not see their physician, and they both could not afford health care.” Dying with dignity was often expressed within this theme, including prior conversations with health care providers, “one victim discussed ‘dying with dignity’ with their health care provider. [T]hem and their partner were both of sound mind, but failing bodies, and did not see a point in prolonging the inevitable.”

### Social factors of despair

We also identified situational factors as contributing to rural multiple‐death suicides including financial strain, legal problems, or interpersonal violence. For example, some of these situations included decedents with “a history of drug abuse, were known to local authorities for domestic violence, and were served with eviction papers due to unpaid rent.” We also identified situations of sexual violence and bullying, where decedents were both reportedly victims of interpersonal violence as well as those who were accused of being perpetrators (e.g., “both decedents had recently been accused of molesting a family member”; “victim 1's suicide note stated that they were being bullied at school, and victim 2's suicide note stated that their family did not approve of their homosexual lifestyle”).

## DISCUSSION

In this national, mixed‐methods study, we identified four themes for multiple‐death suicides among rural US populations. We observed that, though rare, commonly occurring situations for multiple‐death suicides in the rural United States included older adults who were married and had chronic health conditions or social challenges. Most of these suicides were also planned prior to their deaths and involved providing notes to their next of kin.

Decedents were less likely to disclose intentions of suicides to others and have mental health problems, suggesting that decedents who die from multiple‐death suicide may show less signs of suicidal behaviors and were more likely to have planned privately with their intimate partner. Previous studies on multiple‐death suicides have suggested that these deaths are less often impulsive and were planned prior to their deaths.^15^, ^16^ However, our study provides a more detailed description of these multiple‐death suicides including that they tended to be older, married couples dying at their own discretion as opposed to dying later in life. Our study also suggested a desire to die with an intimate partner rather than dying alone. Previous literature on the prevailing theory of suicide (i.e., the interpersonal theory of suicide) suggests one necessary component for suicide to occur includes a lack of self‐preservation from relentless suicidal ideation and suicide planning.^24^, ^25^ In 2021, over 20% of suicide decedents were known to have discussed their intent for suicide with someone, of which, approximately 39% were former or current intimate partners, approximately 37% were other family members, and approximately 14% were friends or colleagues.^26^ Therefore, suicide prevention strategies should not only consider an individual at higher risk for suicide but also their intimate partners, family members, and close relationships.

Having unbearable health conditions like chronic pain and cancer were also common among multiple‐death suicides in the rural United States. While the prevalence of physical health problems was not different between multiple‐death suicides and single suicides in the rural United States, this may suggest physical health problems are an issue for both types of suicide in the rural United States. Previous studies have observed that chronic health conditions are associated with suicide, especially among older adults.^27^, ^28^, ^29^ We also found that decedents would report these health conditions to be unbearable and that they could no longer manage these conditions or see their intimate partner live with these chronic conditions. Our finding aligns with previous literature that suggests suicidality is exacerbated when chronic conditions coincide with other functional impairments including the capacity to manage stress and chronic pain^30^, ^31^, ^32^ and stressful life events (e.g., financial burdens, legal problems).^30^, ^32^ Use of suicide risk assessments for older adults in chronic disease clinical care settings may be a potential point of suicide prevention intervention.^30^, ^33^ Previous literature has suggested older adults were at the highest risk for suicidal ideation in the earliest stages of chronic disease treatment course.^30^, ^31^, ^32^ However, our results differed from previous literature that had suggested that suicide risk was highest during early treatment stages,^30^ meaning that testing for suicidality should be conducted at all stages of chronic disease progression. An additional setback to intervening through clinical care is the limited access to health care in the rural United States.^6^, ^13^, ^22^, ^34^, ^35^ Improving access to care through options like telemedicine have been shown to improve access to care for suicidal ideation.^9^ However, future studies should also examine how improving access to treatment and management of chronic conditions may reduce multiple‐death suicides and single suicides in the rural United States.

We also identified that social factors including financial problems, criminal legal problems, and being victims of violence may contribute to multiple‐death suicides in the rural United States. While the odds of multiple‐death suicides were lower for these social factors, this is likely due to small cell counts. Of the social factors identified, most involved some form of financial strain. Previous studies have suggested suicides—and other deaths are despair—are higher among rural US populations due, in part, to economic burdens, especially among those who are of a lower socioeconomic status.^6^, ^27^, ^29^, ^36^, ^37^, ^38^, ^39^, ^40^, ^41^, ^42^ Improving social safety nets including unemployment programs and providing better access to health insurance and affordable housing have been shown to reduce suicide,^6^, ^43^ which may reduce multiple‐death suicides.

### Strengths and limitations

This study has strengths. This is the first study to examine themes and factors related to multiple‐death suicides in the rural United States. Additionally, we used NVDRS, which includes all 50 states, the District of Columbia, and Puerto Rico, suggesting that the findings are generalizable to all rural US populations. Finally, we used a mixed‐methods approach to validate the themes we had identified in our content analysis and provide additional context for the different factors that contribute to multiple‐death suicides.

This study has limitations. Because there were only 99 suicide decedents in the multiple‐death suicide group, findings from the quantitative analysis have limited statistical power. However, the results tend to align with the themes identified from the qualitative analysis. Moreover, we used RUCCs to determine rurality of decedents, which may potentially misclassify decedents as urban when they live in rural areas within an urban county. Unfortunately, RUCCs were the best measure available that could be linked to suicide decedents in NVDRS. Finally, the qualitative portion uses summaries of CME/LE reports, which may not adequately reflect all circumstances in a decedent's life (e.g., a decedent's religious affiliation) and provide an inherently biased view. Hence, the findings should be considered with this understanding.

## CONCLUSIONS

We found several differences between multiple‐death suicides and single suicides in the rural United States including the involvement of older and married adults and those with unbearable health conditions, suggesting that different prevention methods may need to be considered for multiple‐death suicides. Future research should assess the impacts of providing better access to chronic disease treatment and management on multiple‐death suicides in the rural United States.

## CONFLICT OF INTEREST STATEMENT

The authors declare no conflicts of interest.

## Data Availability

Data from the National Violent Death Reporting System Restricted Access Database are available upon request from the Centers for Disease Control and Prevention.

## APPENDIX A CODEBOOK OF DOMAINS, CODES, AND DEFINITIONS.

**Table:**

Overarching research question	Code	Definition (What it is)	Boundary (What it is not)
What are patterns observed among suicide dyads in rural US counties?	Planned suicide	Narrative notes suicide decedents had described planning the suicide together in the suicide note, from others who knew them, or from officer's suspicion.	Not suicide notes that do not specify plans or from suicide decedents dying in the same location.
	“Domino effect” suicide	Narrative notes suicide decedents did not die together, rather one individual died from suicide and the other individual died following this death.	Not a suicide where it was planned together or where the deaths are unrelated.
	Financial problems	Narrative notes suicide decedent(s) had financial problems contribute to the suicide such as eviction notices and/or problems with keeping up with bills.	Does not include problems with the economy or other types of problems.
	Intimate partner suicide	Narrative notes suicide decedents were married, dating, had a civil union, or romantically involved.	Does not include friends or family members that were not intimate partners.
	Suffering from health conditions	Narrative notes suicide decedent(s) had physical or mental health problems that lead to worse living conditions.	Does not include decedents with diagnoses of health conditions or undiagnosed conditions.
	Legal problems	Narrative notes suicide decedent(s) were facing legal trouble, including time in jail, sentencing, or were suspected of a crime.	Does not include issues solely related to finances, health problems, or anything that does not involve criminal charges.
	Did not want to be a burden	Narrative notes suicide decedent(s) did not want to be a burden family, friends, or others through notes or disclosure to others.	Does not include decedents disclosing suicidal intent without discussing feeling like burdens.
	Victim of violence	Narrative notes suicide decedent(s) were victims of violence such as bullying, abuse, intimate partner violence, or other forms of violence.	Does not include arguments prior to their deaths or being perpetrators of violence.
	Carbon monoxide poisoning	Narrative notes suicide decedents were deceased because of high levels of carbon monoxide, either stated by police or by evidence of high carboxyhemoglobin	Does not include asphyxiation by gases other than Carbon Monoxide.
	Neurodegenerative diseases	Narrative notes suicide decedent(s) were suffering from neurodegenerative diseases, defined as Alzheimer's, Dementia, and Parkinson's disease	Does not include any disease other than these three diseases.
	Location at home	Narrative notes suicide decedent(s) had died by suicide in their home or on their property.	Does not include decedents who died in their car not parked on their property.
	Location outside of home	Narrative notes suicide decedent(s) had died by suicide outside of their home or property.	Does not include individuals who died outside the home but still on the decedent's property.
	Previous self‐harming or suicide attempts	Narrative notes suicide decedent(s) had a history of suicidal ideation, thoughts, or suicide attempts.	Narrative suggests suicidal ideation or disclosure but no previous attempts or self‐harming behaviors
	Previous suicidal disclosure or ideation	The narrative notes suicide decedent(s) disclosed they were going to attempt suicide or felt suicidal to someone else.	Does not include those who disclosed feeling sad or depressed but not suicidal.
	Mental health problems and diagnoses	Narrative notes suicide decedent(s) had mental health problems or were diagnosed with a mental health condition	Does not consider decedents who feel sad or depressed.
	Alcohol on scene, use, or suspected problems	The narrative states that alcohol paraphernalia (e.g., bottles, cans) was found at the scene, decedents were suspected to have used alcohol or alcohol was found in the toxicology screening, or decedents were known to have alcohol problems.	Does not consider drugs other than EtOH.
	Drugs on scene, suspected use, or suspected problems	Narrative states that substance paraphernalia (e.g., pipes) was found at the scene, decedents were suspected to have used substances or substances were found in the toxicology screening, or decedents were known to have substance use problems.	Does not include use or problems related to alcohol or nondrugs like carbon monoxide
	“Could not live without their partner.”	Suicide decedents disclosed to others or wrote a note that they were unable to live without their partners.	Does not include decedents who died at same time as their partner and did not state wanting to die with their partner

## References

[1] Suicide and Self‐Inflicted Injury. National Center for Health Statistics, Centers for Disease Control and Prevention; 2024.

[2] Suicide. National Institute of Mental Health; 2024.

[3] Web‐Based Injury Statistics Query and Reporting System (WISQARS). Centers for Disease Control and Prevention, National Centers for Injury Prevention and Control; 2024.

[4] Suicide in Rural Areas. Rural Health Information Hub; 2024.

[5] Suicide in Rural America as a Public Health Issue. Centers for Disease Control and Prevention (CDC); 2024.

[6] Mohatt NV , Kreisel CJ , Hoffberg AS , Mph LW , Beehler SJ . A systematic review of factors impacting suicide risk among rural adults in the United States. J Rural Health. 2021;37(3):565‐575. 33210399 10.1111/jrh.12532

[7] Kaplan MS , McFarland BH , Huguet N , et al. Acute alcohol intoxication and suicide: a gender‐stratified analysis of the National Violent Death Reporting System. Inj Prev. 2013;19(1):38‐43. 22627777 10.1136/injuryprev-2012-040317 PMC3760342

[8] Kalesan B , Zhao S , Poulson M , et al. Intersections of firearm suicide, drug‐related mortality, and economic dependency in rural America. J Surg Res. 2020;256:96‐102. 32688080 10.1016/j.jss.2020.06.011

[9] Tarlow KR , Johnson TA , McCord CE . Rural status, suicide ideation, and telemental health: risk assessment in a clinical sample. J Rural Health. 2019;35(2):247‐252. 29940082 10.1111/jrh.12310

[10] Rural Behavioral Health: Telehealth Challenges and Opportunities. Substance Abuse and Mental Health Services Administration (SAMHSA); 2016.

[11] Morales DA , Barksdale CL , Beckel‐Mitchener AC . A call to action to address rural mental health disparities. J Clin Transl Sci. 2020;4(5):463‐467. 33244437 10.1017/cts.2020.42 PMC7681156

[12] Monteith LL , Holliday R , Brown TL , Brenner LA , Mohatt NV . Preventing suicide in rural communities during the COVID‐19 pandemic. J Rural Health. 2021;37(1):179‐184. 32282968 10.1111/jrh.12448 PMC7262063

[13] Searles VB , Valley MA , Hedegaard H , Betz ME . Suicides in urban and rural counties in the United States, 2006–2008. Crisis. 2014;35(1):18‐26. 24067250 10.1027/0227-5910/a000224

[14] National Violent Death Reporting System Web Coding Manual Version 6. Centers for Disease Control and Prevention, National Center for Injury Prevention and Control; 2022.

[15] Kim KV , Russell C , Kaplan MS , Rehm J , Lange S . Types of suicide pacts: a comparative analysis using the National Violent Death Reporting System. Front Psychiatry. 2023;14:1139305. 37215672 10.3389/fpsyt.2023.1139305 PMC10196347

[16] Ashley J , Kim KV , Russell C , Lange S . A comparative analysis of solitary suicides, suicides following homicide, and suicide pacts using the National Violent Death Reporting System. BMC Psychiatry. 2023;23(1):1. 36593442 10.1186/s12888-022-04495-w PMC9808963

[17] Ballesteros MF , Ivey‐Stephenson AZ , Trinh E , Stone DM . Background and rationale—CDC guidance for communities assessing, investigating, and responding to suicide clusters, United States, 2024. MMWR Suppl. 2024;73(2):1‐7. 10.15585/mmwr.su7302a1 PMC10899087 38412112

[18] Institute of Medicine , National Research Council . Contagion of Violence: Workshop Summary . National Academies Press; 2013. 24649515

[19] Rural–Urban Continuum Codes. United States Department of Agriculture (USDA) Economic Research Service. Accessed June 1, 2024. https://www.ers.usda.gov/data‐products/rural‐urban‐continuum‐codes/

[20] National Violent Death Reporting System User Guidelines. Mortality Surveillance Team, Surveillance Branch, Division of Violence Prevention, National Center for Injury Prevention & Control, Centers for Disease Control and Prevention; 2024.

[21] Dedoose Version 9.2.12, cloud application for managing, analyzing, and presenting qualitative and mixed method research data. SocioCultural Research Consultants, LLC; 2024.

[22] Perry SW , Rainey JC , Allison S , et al. Achieving health equity in US suicides: a narrative review and commentary. BMC Public Health. 2022;22(1):1360. 35840968 10.1186/s12889-022-13596-w PMC9284959

[23] Louis KS , Barton AC . Tales from the science education crypt: a critical reflection of positionality, subjectivity, and reflexivity in research. Forum Qual Soc Res. 2002;3(3):249‐262.

[24] Chu C , Buchman‐Schmitt JM , Stanley IH , et al. The interpersonal theory of suicide: a systematic review and meta‐analysis of a decade of cross‐national research. Psychol Bull. 2017;143(12):1313‐1345. 29072480 10.1037/bul0000123 PMC5730496

[25] Van Orden KA , Witte TK , Cukrowicz KC , Braithwaite SR , Selby EA . The interpersonal theory of suicide. Psychol Rev. 2010;117(2):575‐600. 20438238 10.1037/a0018697 PMC3130348

[26] Nguyen BL , Lyons BH , Forsberg K , et al. Surveillance for Violent Deaths—National Violent Death Reporting System, 48 States, the District of Columbia, and Puerto Rico, 2021. MMWR Surveill Summ. 2024;73(5):1‐44. 10.15585/mmwr.ss7305a1 PMC11262823 38980822

[27] Steele IH , Thrower N , Noroian P , Saleh FM . Understanding suicide across the lifespan: a united states perspective of suicide risk factors, assessment & management. J Forensic Sci. 2018;63(1):162‐171. 28639299 10.1111/1556-4029.13519

[28] Massetti GM , Holland KM , Jack SPD , Ragan KR , Lunsford NB . Circumstances of suicide among individuals with a history of cancer. Psychooncology. 2018;27(7):1750‐1756. 29624792 10.1002/pon.4720 PMC6063079

[29] Petrosky E , Harpaz R , Fowler KA , et al. Chronic pain among suicide decedents, 2003 to 2014: findings from the national violent death reporting system. Ann Intern Med. 2018;169(7):448‐455. 30208405 10.7326/M18-0830 PMC6913029

[30] Raue PJ , Ghesquiere AR , Bruce ML . Suicide risk in primary care: identification and management in older adults. Curr Psychiatry Rep. 2014;16(9):466. 25030971 10.1007/s11920-014-0466-8 PMC4137406

[31] Waern M , Rubenowitz E , Runeson B , Skoog I , Wilhelmson K , Allebeck P . Burden of illness and suicide in elderly people: case‐control study. BMJ. 2002;324(7350):1355. 12052799 10.1136/bmj.324.7350.1355 PMC115206

[32] Conwell Y , Thompson C . Suicidal behavior in elders. Psychiatr Clin North Am. 2008;31(2):333‐356. 18439452 10.1016/j.psc.2008.01.004 PMC2735830

[33] Schulberg HC , Bruce ML , Lee PW , Williams JW Jr , Dietrich AJ . Preventing suicide in primary care patients: the primary care physician's role. Gen Hosp Psychiatry. 2004;26(5):337‐345. 15474633 10.1016/j.genhosppsych.2004.06.007

[34] Brundisini F , Giacomini M , DeJean D , Vanstone M , Winsor S , Smith A . Chronic disease patients' experiences with accessing health care in rural and remote areas: a systematic review and qualitative meta‐synthesis. Ont Health Technol Assess Ser. 2013;13(15):1‐33. PMC3817950 24228078

[35] Disler R , Pascoe A , Hickson H , et al. Service level characteristics of rural palliative care for people with chronic disease. J Pain Symptom Manage. 2023;66(4):301‐309. 37343902 10.1016/j.jpainsymman.2023.06.003

[36] Alexopoulos EC , Kavalidou K , Messolora F . Suicide mortality patterns in Greek work force before and during the economic crisis. Int J Environ Res Public Health. 2019;16(3):469. 30736267 10.3390/ijerph16030469 PMC6388265

[37] Peek‐Asa C , Zhang L , Hamann C , Davis J , Schwab‐Reese L . Characteristics and circumstances associated with work‐related suicides from the national violent death reporting system, 2013–2017. Int J Environ Res Public Health. 2021;18(18):9538. 34574474 10.3390/ijerph18189538 PMC8465410

[38] Case A , Deaton A . Mortality and morbidity in the 21(st) century. Brookings Pap Econ Act. 2017;2017:397‐476. 29033460 10.1353/eca.2017.0005 PMC5640267

[39] Case A , Deaton A . Rising morbidity and mortality in midlife among white non‐Hispanic Americans in the 21st century. Proc Natl Acad Sci USA. 2015;112(49):15078‐15083. 26575631 10.1073/pnas.1518393112 PMC4679063

[40] Luo F , Florence CS , Quispe‐Agnoli M , Ouyang L , Crosby AE . Impact of business cycles on US suicide rates, 1928–2007. Am J Public Health. 2011;101(6):1139‐1146. 21493938 10.2105/AJPH.2010.300010 PMC3093269

[41] Fowler KA , Gladden RM , Vagi KJ , Barnes J , Frazier L . Increase in suicides associated with home eviction and foreclosure during the US housing crisis: findings from 16 National Violent Death Reporting System States, 2005–2010. Am J Public Health. 2015;105(2):311‐316. 25033148 10.2105/AJPH.2014.301945 PMC4318323

[42] Webb RT , Qin P , Stevens H , Mortensen PB , Appleby L , Shaw J . National study of suicide in all people with a criminal justice history. Arch Gen Psychiatry. 2011;68(6):591‐599. 21300938 10.1001/archgenpsychiatry.2011.7

[43] Stone DM , Holland KM , Bartholow B , Crosby AE , Davis S , Wilkins N . Preventing Suicide: A Technical Package of Policies, Programs, and Practices. National Center for Injury Prevention and Control, Centers for Disease Control and Prevention; 2017.

